# Peripheral Schwannoma Presenting as a Retro-Malleolar Mass: A Case Report

**DOI:** 10.7759/cureus.42137

**Published:** 2023-07-19

**Authors:** Lokeshwar Raaju Addi Palle, Venu Gopal Reddy Depa, Krushi Shah, Cuauhtemoc Jeffrey Soto, Bitania A Aychilluhim, Vipul V Rakhunde

**Affiliations:** 1 Department of Surgery, Kamala Children's Hospital, Chennai, IND; 2 Department of Internal Medicine, Manthena Narayana Raju (MNR) Medical College and Hospital, Sangareddy, IND; 3 Department of Internal Medicine, Gujarat Medical Education and Research Society (GMERS) Medical College, Gandhinagar, IND; 4 Department of Research and Development, Universidad Juárez del Estado de Durango, Durango, MEX; 5 Department of Internal Medicine, California Institute of Behavioral Neurosciences and Psychology, Fairfield, USA; 6 Department of General Practice, Learn and Live (L&L) Wholestic Health Services, Lincolnia, USA; 7 Department of Internal Medicine, Hallelujah General Hospital, Addis Ababa, ETH; 8 Department of Cardiology, Paras Hospitals, Panchkula, IND

**Keywords:** treatment, differential diagnosis, surgical excision, paresthesias, pain, numbness, plantar foot, case report, peripheral schwannoma

## Abstract

Schwannomas are rare peripheral nerve tumors that can present with diverse clinical manifestations. They commonly present as solitary, encapsulated masses and can occur in various locations throughout the body. This case report presents a comprehensive analysis of a peripheral schwannoma in a 29-year-old male patient who presented with numbness, pain, and paresthesias on the plantar aspect of the left foot. The symptoms progressively worsened, impacting the patient's daily activities. Physical examination revealed tenderness on the medial aspect of the left foot, along with prolonged episodes of paresthesia and recurrent numbness. Imaging studies confirmed the presence of a retro-malleolar mass, consistent with a peripheral schwannoma. The patient underwent successful surgical excision of the mass, resulting in complete resolution of symptoms. This case emphasizes the importance of considering peripheral schwannomas in the differential diagnosis of patients presenting with foot symptoms and highlights the effectiveness of surgical excision as a treatment modality for these tumors.

## Introduction

Schwannomas, also known as neurilemmomas, are benign tumors originating from the Schwann cells that surround the sheaths of peripheral, cranial, or autonomic nerves. These encapsulated tumors develop when proliferating Schwann cells form a tumor around the nerve sheath [1]. Schwannomas affect both males and females equally, with the highest incidence occurring between the ages of 30 and 40 years. While they are commonly localized in the skin or subcutaneous tissue, their occurrence in the foot is rare [2]. The annual incidence of peripheral schwannomas is relatively low at 0.6 per 100,000 people, with the majority of cases found on the flexor surfaces of the limbs [3]. They account for approximately 5% of all benign soft tissue neoplasms. Although malignant transformation is extremely rare, distinguishing schwannomas from malignant peripheral nerve sheath tumors can be challenging. Etiological factors such as trauma, Carney's complex, and neurofibromatosis (NF) type 1 or 2 may contribute to the development of these tumors [2].

Standard X-rays typically yield negative results for soft tissue tumors. Ultrasonography and magnetic resonance imaging (MRI) play crucial roles in diagnosing schwannomas, particularly in evaluating superficially located tumors. Schwannomas generally display an iso-intense signal or reduced signal intensity compared to skeletal muscle on T1-weighted images, whereas they demonstrate heterogeneously increased signal intensity on T2-weighted images. Moreover, magnetic resonance imaging helps determine the size, shape, edge, surrounding edema of the lesion, invasion of adjacent blood nerves, and changes in signal intensity, aiding in accurate diagnosis [3,4].

The decision to perform surgery for benign neurogenic tumors should be based on a careful assessment of the risks and benefits. Surgical removal is essential in cases of large tumors that affect major peripheral nerves in the extremities and when there are accompanying symptoms of compression. In the foot, even small schwannomas affecting the plantar aspect can cause symptoms during walking and jumping. Surgical treatment usually includes the removal of the lesion through excision or intracapsular enucleation after making an incision in the epineurium. This surgical approach enables the preservation of the parent nerve and its neurological function, facilitated by the schwannoma's eccentric location [2]. This case report describes a unique presentation of tibial schwannoma as a retro-malleolar mass in a 29-year-old male. The aim is to discuss the clinical presentation, diagnostic workup, treatment approach, and differential diagnosis of peripheral schwannomas.

## Case presentation

A 29-year-old male patient with no significant past medical history presented to the orthopedic department of a tertiary care hospital with a six-month history of numbness, pain, and paresthesias affecting the plantar aspect of his left foot. The symptoms gradually worsened over time, significantly impacting his daily activities. The patient reported experiencing pain specifically while walking on the medial aspect of his left foot. Additionally, the paresthesia and numbness episodes were lasting longer and occurring with increasing frequency.

On examination of the left foot, a localized swelling was observed behind the medial malleolus, which was firm and non-tender to palpation with a positive Tinel test. The skin appeared normal, without any discoloration or visible lesions. The patient reported numbness and paresthesia in the distribution of the left tibial nerve, extending to the sole, heel, and toes. Sensory testing with a monofilament confirmed decreased sensation in the affected areas compared to the unaffected foot. Motor examination was suggestive of normal findings, completely. The patient demonstrated good balance and coordination during gait and balance assessment. Vascular examination revealed palpable dorsalis pedis and posterior tibial pulses, indicating normal arterial blood supply to the foot.

The initial differential diagnoses considered for the patient's clinical manifestations included peripheral neuropathy, tarsal tunnel syndrome, Morton's neuroma, lipoma, ganglion cyst, vascular malformation, and posterior tibial schwannoma.

To further investigate the patient's condition, various diagnostic modalities were employed. Ultrasound imaging revealed an oval-shaped hypoechoic solid mass measuring 3.1 × 1.6 cm near the medial malleolus, raising suspicion of a benign peripheral nerve sheath tumor (color Doppler was also done to rule out any vascular involvement). Fine needle aspiration cytology (FNAC) was performed under ultrasound guidance to obtain tissue samples from the mass. Four unstained slides, including two Giemsa and two Pap-stained slides, were prepared for microscopic examination. Microscopic examination of the smears revealed scattered fragments of a mesenchymal neoplasm consisting of fusiform spindle cells. The cells exhibited elongated, darkly stained nuclei and pale cytoplasm. Notably, no mitotic activity, cytologic atypia, or necrosis was observed. These findings were consistent with a spindle cell neoplasm.

**Figure 1:**
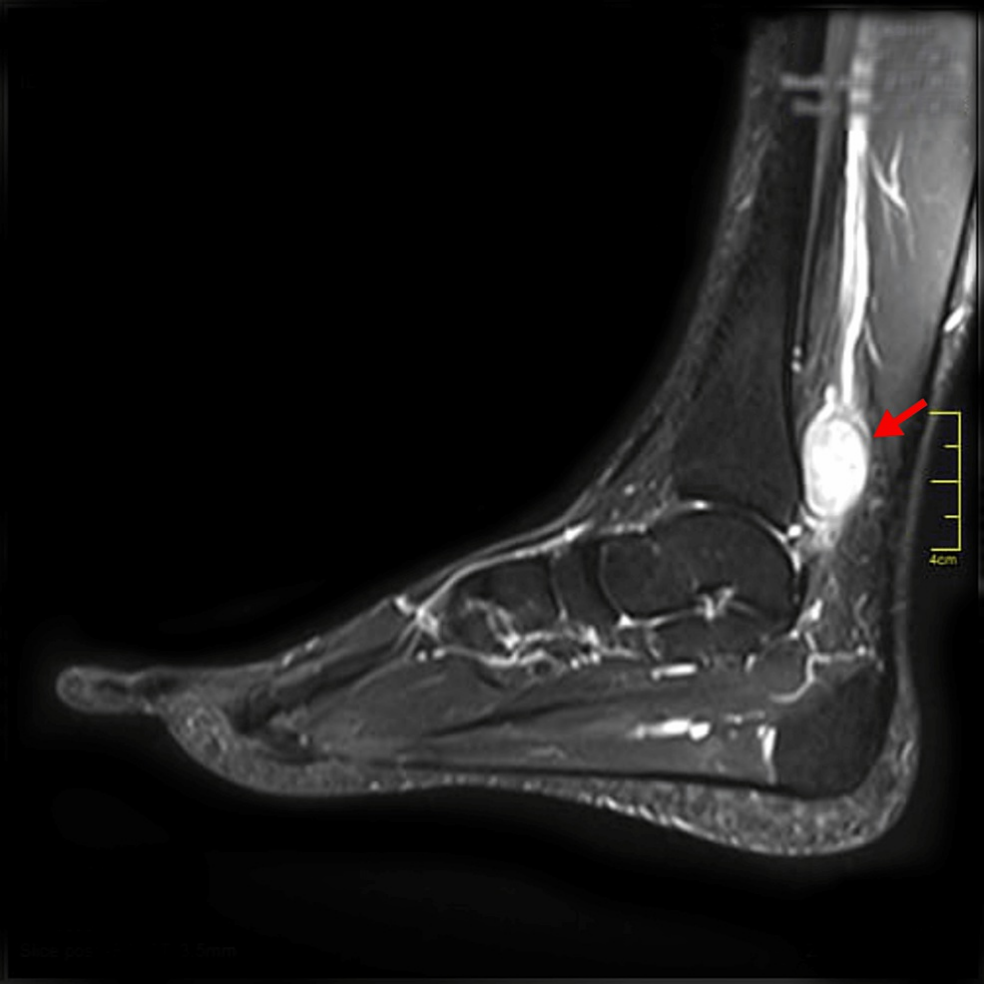
Magnetic resonance imaging (sagittal view) with contrast of the patient's left foot showing hyperintense ovoid mass (red arrow)

Magnetic resonance imaging (MRI) with contrast revealed a 19 × 20 × 28 mm intensely enhancing solid ovoid lesion over the posterior aspect of the distal tibia along the posterior tibial neurovascular bundle, approximately 39 mm from the superior surface of the calcaneus (Figure 1).

**Figure 2:**
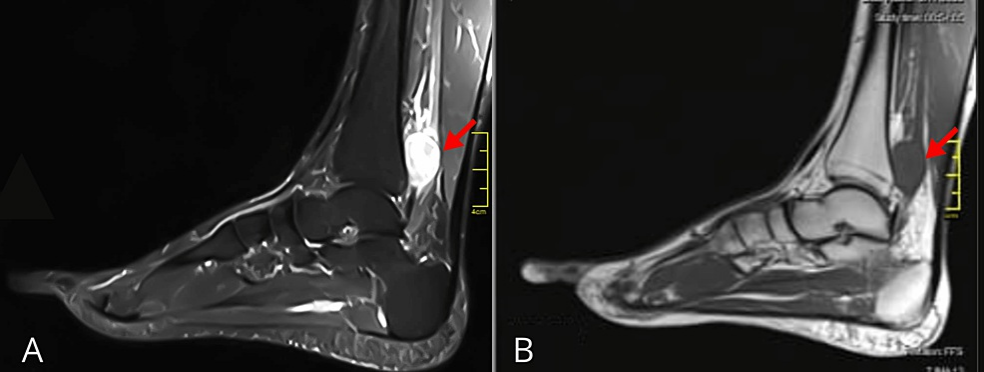
Magnetic resonance imaging (sagittal view) of the patient exhibiting heterogeneously increased signal intensity on T2-weighted image (A) while showing iso-intensity on T1-weighted image (B) of the foot (red arrow)

The schwannoma appeared iso-intense on T1-weighted images while exhibiting heterogeneously increased signal intensity on T2-weighted images (Figure 2). There is no underlying marrow edema. No other significant pathology was observed on the scan. These radiological findings were consistent with a nerve sheath tumor, confirming the diagnosis of tibial schwannoma.

**Figure 3:**
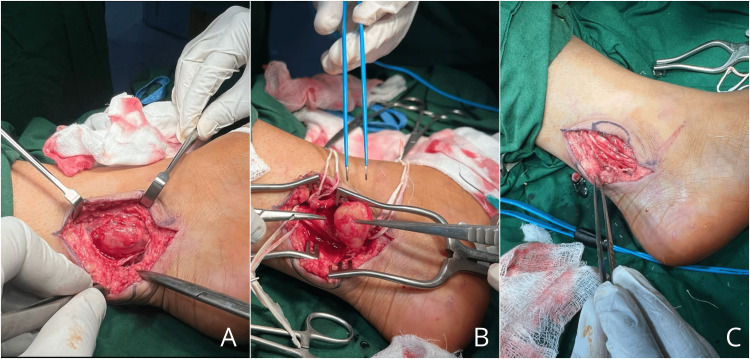
Intraoperative images showing the dissected tibial nerve schwannoma in the left retro-malleolar area (A) and complete excision of schwannoma from fascicles of the tibial nerve (B,C)

Considering the clinical progression and radiological findings, the decision was made to proceed with total surgical excision of the mass. The procedure was performed under general anesthesia using a medial approach. The posterior tibial nerve was identified, revealing the presence of the mass within it. The use of loupe magnification enabled meticulous dissection and complete excision of the mass (Figure 3). The procedure was successfully performed, resulting in symptom relief and preservation of neurological function.

**Figure 4:**
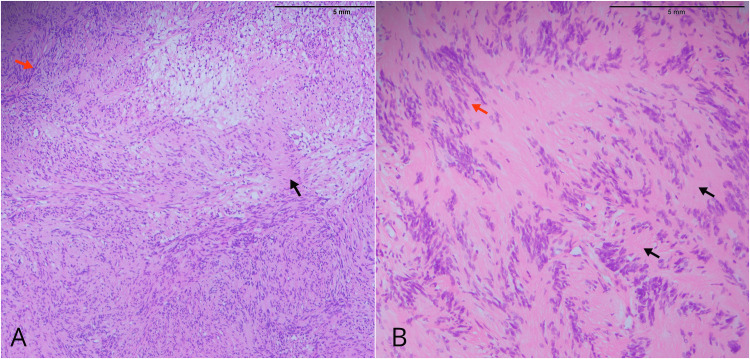
Tibial nerve sheath tumor revealed a well-circumscribed, encapsulated lesion composed of spindle cells on hematoxylin and eosin staining (A) 10× magnification, (B) 40× magnification Black arrows show Verocay bodies. Red arrows show the palisading pattern.

The histopathological examination of the specimen revealed a well-circumscribed, encapsulated lesion composed of spindle cells. The tumor exhibited areas with varying cellularity, including hypocellular and hypercellular regions. The hypercellular areas displayed minimal cellular atypia and the presence of Verocay bodies, characteristic of schwannomas. Anaplastic features were not identified. Based on these findings, a diagnosis of tibial schwannoma was confirmed (Figure 4).

The patient underwent regular follow-up visits at six months post-surgical excision of the tibial schwannoma. During the follow-up examination, the patient reported complete resolution of the previously experienced symptoms, including numbness, pain, and paresthesias in the left foot. The localized swelling behind the medial malleolus had completely resolved.

## Discussion

Schwannomas, also known as neurilemmomas, represent 8% of all primary brain tumors, with 80%-90% of cases occurring in the cerebellopontine angle. While symptomatic schwannomas in the lower extremity are rare, their occurrence in the foot and ankle region is particularly uncommon. Reported cases range from 1% to 10%. In a comprehensive review conducted by Kim et al., it was revealed that among 397 cases of peripheral nerve sheath tumors, only a small proportion of 32 cases (8.86%) were specifically identified as schwannomas situated in the lower extremity. Another study by Odom et al. estimated the prevalence of schwannomas in the foot to be as low as 2.93% [3].

The onset of symptoms is associated with progressive tumor growth and the development of a sufficiently large tumor volume that compresses the originating nerve. Prolonged compression initially leads to venous insufficiency, causing fatigue, discomfort, and eventually pain in the affected area. Mechanical compression also disrupts axonal transport, exacerbating nerve dysfunction and resulting in structural damage, axonolysis, and Wallerian degeneration [4-6]. Clinically, schwannomas present as oval masses with firm consistency, eccentric to the nerve. Schwannomas commonly exhibit dimensions below 3 cm in diameter, possessing a well-defined capsule. They demonstrate a slow growth rate, lack invasive tendencies, and present a smooth surface with a coloration ranging from yellowish-gray to whitish-gray. Initial symptoms may include painless edema persisting for years, followed by pain, paresthesia, hypoesthesia, and motor deficits as adjacent structures are compressed and local tissue lacks distensibility [7]. Although schwannomas are usually solitary (95%), multiple tumors can occur in the extremities, a condition referred to as schwannomatosis. Schwannomas exhibit slow and non-infiltrative growth patterns, often presenting as painless swellings without specific symptoms. Neurological symptoms, such as neuropathic pain, dysesthesia, sensory loss, and tingling sensations, commonly occur when schwannomas exceed 25 mm in diameter and exert pressure on the originating nerve [6]. Schwannomas are mostly sporadic but may be associated with neurofibromatosis type 2 (NF2), schwannomatosis, or Carney's complex [5].

In terms of diagnostic modalities, ultrasonography typically reveals solid, sharply delineated, ovoid, hypoechoic homogenous masses. X-rays may be performed to rule out any bone involvement or abnormalities. Further evaluation can be conducted using magnetic resonance imaging (MRI) with gadolinium contrast. Schwannomas generally display an iso-intense signal or reduced signal intensity compared to skeletal muscle on T1-weighted images, whereas they demonstrate heterogeneously increased signal intensity on T2-weighted images. Certain imaging features such as the "fascicular sign," "target sign," and "split fat sign" may be observed, although some of these signs can also be seen in neurofibromas. Large schwannomas with heterogeneous appearances on MRI can be distinguished from malignant peripheral nerve sheath tumors by analyzing the imaging features at and near the margin of the mass. The presence of the split fat sign and bright rim sign, along with the absence of a lobular shape and extensive edema, favors the diagnosis of schwannoma, particularly when two or more of these imaging features are present [3,8]. Ultrasonography and MRI are effective means of detecting and characterizing schwannomas, while normal X-rays are usually inadequate for their visualization [2]. Additionally, magnetic resonance imaging helps determine the size, shape, edge, surrounding edema of the lesion, invasion of adjacent blood nerves, and changes in signal intensity, aiding in accurate diagnosis [4].

Histopathologically, schwannomas can be classified into two distinct types known as Antoni A and Antoni B. Antoni A primarily consists of spindle cells with ill-defined boundaries, arranged in leaves or bundles, and exhibiting wavy nuclei. Mitotic figures are sparsely distributed. Verocay bodies, characterized by palisade arrangements of cells with areas devoid of nuclei between them, are also observed. On the other hand, Antoni B regions are less cellular and lack distinct architectural features. The matrix in Antoni B areas contains a mixture of spindle and oval cells, numerous blood vessels, sparse collagen, and a variety of inflammatory cells, including histiocytes. Some histiocytes within these regions may contain hemosiderin. Degenerative changes, such as hemorrhage, vessel wall hyalinization, cyst formation, fibrin deposition, focal calcification, and matrix fibrosis, are commonly observed. Schwann cell nuclei become hyperchromatic and multilobulated, resulting in a grayish-yellow coloration. Immunohistochemical tests show positive immunoreactivity for the S-100 and Leu-7 proteins [7].

Treatment modalities for major nerve schwannomas depend on the patient's symptoms. Asymptomatic patients with confirmed diagnoses through MRI are typically managed conservatively. However, it has been cautioned that larger tumors carry a higher risk of major neurological deficits following surgery, suggesting that early surgical enucleation is strongly recommended when a schwannoma is detected. Surgical enucleation is the established treatment modality, although some schwannomas may pose challenges for complete enucleation, potentially leading to iatrogenic nerve injuries despite atraumatic procedures. Adequate exposure of the affected nerve, with sufficient proximal and distal margins, is crucial for precise tumor visualization, intraoperative nerve stimulation, and monitoring. The use of intraoperative magnifying glasses and gentle nerve manipulation helps preserve nerve continuity and function. Patients should be informed about the possibility of transient partial sensory or motor function loss in the affected nerve region [2].

The diagnosis of schwannoma in the lower limb often experiences a delay of several years due to frequent misdiagnosis as a benign solitary mass, such as a myxoma, fibroma, or ganglion [4]. The primary differential diagnoses include neurofibroma, synovial cyst, low-flow venous malformations, and high-grade sarcomas such as fibrosarcoma, synovial sarcoma, or leiomyosarcoma. Magnetic resonance imaging with gadolinium contrast is valuable in distinguishing between schwannomas and synovial cysts, particularly when the associated nerve is not visualized. Neurofibromatosis type 1 (NF1) is characterized by the presence of plexiform neurofibromas, which typically manifest at birth or within a few months after birth. Considering the potential for neurofibromas to undergo malignant transformation, it is essential to consider differential diagnoses. Previous studies have identified specific MRI features that assist in differentiating between schwannomas and neurofibromas, although a definitive diagnosis should be confirmed through histological examination, especially in cases of the plexiform variant. Indolent growth or symptoms may be observed in high-grade sarcomas of the extremities, leading to a resemblance to benign soft tissue lesions. However, it is recommended to perform ultrasonography-guided biopsies during the definitive surgical approach, with excisional biopsies avoided whenever possible [2]. Differentiating schwannomas from ganglion cysts can be challenging, as they often present similar clinical symptoms and the accumulation of cystic fluid in the adjacent articular space [4].

Schwannomas typically exhibit slow growth and manifest as painless swelling that persists for several years without specific symptoms. The diagnosis of a schwannoma in a lower limb is often delayed due to frequent misdiagnosis as a benign solitary mass, such as a ganglion, fibroma, or myxoma. Recurrence is extremely rare, occurring in less than 1% of cases, unless there is incomplete excision of the tumor [9]. Malignant transformation of peripheral nerve schwannomas is highly uncommon. Therefore, the management of peripheral schwannomas focuses on providing pain relief while minimizing the risk of additional neurological deficits [5].

Schwannoma of the foot is a rare neoplasm that affects both males and females equally. Histologically, it is characterized by the presence of palisaded spindle cells forming distinct Antoni A and B zones. Ultrasonography and magnetic resonance imaging play crucial roles in diagnosing schwannomas, providing valuable information regarding tumor location, size, composition, involvement of adjacent nerves, and relationships with surrounding muscular structures. Surgical excision is the primary treatment modality, and recurrence is infrequent [9].

## Conclusions

This case report highlights the clinical presentation, diagnostic workup, and successful treatment of a peripheral schwannoma involving the plantar aspect of the left foot. Peripheral schwannomas should be considered in the differential diagnosis of patients presenting with foot symptoms, and appropriate imaging studies, such as ultrasound and MRI, can aid in the diagnosis. Surgical excision remains the primary treatment modality, leading to favorable outcomes and symptom resolution. Healthcare professionals should be aware of peripheral schwannomas as a potential cause of foot symptoms, ensuring timely diagnosis and appropriate management.

## References

[1] Sitenga JL, Aird GA, Nguyen A, Vaudreuil A, Huerter C (2017). Clinical features and surgical treatment of schwannoma affecting the base of the tongue: a systematic review. Int Arch Otorhinolaryngol.

[2] Angelini A, Bevoni R, Biz C, Cerchiaro MC, Girolami M, Ruggieri P (2019). Schwannoma of the foot: report of four cases and literature review. Acta Biomed.

[3] Majumder A, Ahuja A, Chauhan DS, Paliwal P, Bhardwaj M (2021). A clinicopathological study of peripheral schwannomas. Med Pharm Rep.

[4] Zhang Y, Wei Z, Zhang G, Wang D (2022). Sciatic nerve schwannoma in the lower limb mimicking ganglion cyst. Am J Phys Med Rehabil.

[5] El Sayed L, H Masmejean E, Lavollé A, Biau D, Peyre M (2022). Clinical results after surgical resection of benign solitary schwannomas: a review of 150 cases. Orthop Traumatol Surg Res.

[6] Muramatsu K, Tani Y, Seto T, Iwanaga R, Mihara A, Ihara K, Sakai T (2021). Schwannoma in the extremity: clinical features and microscopic intra-capsular enucleation. J Rural Med.

[7] Galbiatti JA, Milhomens GR, Bertozzo LG, Escames L, Milhomens Neto PA, Galbiatti MG (2020). Retrospective analysis of 20 patients affected by schwannomas in the upper and lower limbs. Rev Bras Ortop (Sao Paulo).

[8] Zhang Z, Deng L, Ding L, Meng Q (2015). MR imaging differentiation of malignant soft tissue tumors from peripheral schwannomas with large size and heterogeneous signal intensity. Eur J Radiol.

[9] Hao X, Levine D, Yim J (2019). Schwannoma of foot and ankle: seven case reports and literature review. Anticancer Res.

